# Remote Sensing Analysis of Vegetation Recovery following Short-Interval Fires in Southern California Shrublands

**DOI:** 10.1371/journal.pone.0110637

**Published:** 2014-10-22

**Authors:** Ran Meng, Philip E. Dennison, Carla M. D’Antonio, Max A. Moritz

**Affiliations:** 1 Department of Geography, University of Utah, Salt Lake City, Utah, United States of America; 2 Department of Ecology, Evolution and Marine Biology, University of California Santa Barbara, Santa Barbara, California, United States of America; 3 Department of Environmental Science, Policy, and Management, University of California, Berkeley, California, United States of America; DOE Pacific Northwest National Laboratory, United States of America

## Abstract

Increased fire frequency has been shown to promote alien plant invasions in the western United States, resulting in persistent vegetation type change. Short interval fires are widely considered to be detrimental to reestablishment of shrub species in southern California chaparral, facilitating the invasion of exotic annuals and producing “type conversion”. However, supporting evidence for type conversion has largely been at local, site scales and over short post-fire time scales. Type conversion has not been shown to be persistent or widespread in chaparral, and past range improvement studies present evidence that chaparral type conversion may be difficult and a relatively rare phenomenon across the landscape. With the aid of remote sensing data covering coastal southern California and a historical wildfire dataset, the effects of short interval fires (<8 years) on chaparral recovery were evaluated by comparing areas that burned twice to adjacent areas burned only once. Twelve pairs of once- and twice-burned areas were compared using normalized burn ratio (NBR) distributions. Correlations between measures of recovery and explanatory factors (fire history, climate and elevation) were analyzed by linear regression. Reduced vegetation cover was found in some lower elevation areas that were burned twice in short interval fires, where non-sprouting species are more common. However, extensive type conversion of chaparral to grassland was not evident in this study. Most variables, with the exception of elevation, were moderately or poorly correlated with differences in vegetation recovery.

## Introduction

Fire is an important ecological process with dynamic interactions between vegetation, biogeochemical cycles, and human activity [1]–[3]. Dry summer climates make Mediterranean ecosystems, like southern California chaparral, particularly prone to wildfire [4], [5]. Southern California has experienced multiple, massive fires over the past two decades. For example, in October 2003, 300,277 ha (742,000 acres) were burned within one week in the Cedar, Otay and Paradise fires. Four years later many of the same areas were burned in the Harris, Witch and Poomacha fires. Repeat, short-interval fires may push ecosystems into new states, and recently there has been much discussion about disturbance regime thresholds beyond which ecosystem characteristics change dramatically due to a loss of resilience of the vegetation [6]. It has been suggested that in ecosystems dominated by fire adapted species, either very long or very short fire return intervals will drive long term compositional change [7], [8]. Given widespread predictions of increasing fire frequency with ongoing climate change [9], [10], it is important to explore whether altered fire return intervals can drive long term vegetation type change (often referred to as “type conversion”), and which environmental variables predict where such vegetation type change is likely to occur.

Chaparral, commonly found in the mountain areas of coastal California, Baja California and foothills of the Sierra Nevada [11], is dominated by evergreen, sclerophyllous, shrub species that have the ability to reestablish themselves following severe fires [12], [13]. Chaparral species have different regeneration modes following fire, and generally can be grouped into three types: obligate seeders, obligate resprouters and facultative seeders [14]. Obligate seeders can germinate from fire-protected seed banks, obligate resprouters can resprout from fire resistant structures, and a few facultative species combine the two regeneration strategies. Hanes [12] observed that the abundance of obligate seeding species in southern California chaparral tended to decrease with the elevation, likely due to the co-variation between elevation and precipitation.

Landscape factors affecting chaparral regeneration include fire severity, fire history, indigenous life forms, site-specific water availability, latitude and topography [12], [14]–[17]. Temporal factors (e.g. post-fire precipitation in wet season, post-fire temperature in winter) may also impact succession patterns in chaparral shrublands [12], [14], [15]. In general, the growth of chaparral shrubs is fast in the early post-fire years and slows when they become large enough to shade and cover sub-shrub and herbaceous plants that may persist [11]. Within five to seven years after fire, chaparral shrubs may regain dense cover [11], but only after two or three decades does their canopy recover to pre-fire status [12].

Although chaparral communities contain fire-adapted species, alterations in characteristics of fire regimes can make these communities vulnerable to alien plant invasion by reducing the abundance of vulnerable species [13], [18]. Short fire return intervals can prevent obligate seeding species from reaching maturity and producing seed [19]–[24]. Zedler et al. [21] for example, showed that three years after a short (1 year) return interval fire in southern California shrublands, there was a drastic decline in density of an obligate seeding shrub species and large reductions in a resprouting species. Likewise, Keeley and Brennan [13] found that native chaparral woody species recovered well in 4 years after a first fire, but declined after a second fire that occurred with a short interval (3 years or 4 years), with obligate seeding shrubs being the most affected. In contrast, annual species increased and exotic annuals outnumbered native ones at the same sites after repeat fires [13]. Lippitt et al. [17] surveyed the post-fire change in chaparral stands after short interval fires (<5 years, 1977–2003) with the help of remote sensing imagery and a historical wildfire dataset in San Diego County, California, and they concluded that alteration and type conversion could occur under short fire return intervals. While these previous studies examined relatively small areas and short periods following multiple fires, they suggest that short fire return intervals can cause substantial changes in site-scale species composition. In addition to short fire return intervals, other factors, such as grazing and atmospheric nitrogen pollution, may contribute to alien plant invasions in semi-arid shrublands [25], [26].

Extensive “range improvement” practices for increasing the production of herbaceous forage cover for livestock have affected the structure and composition of the western United States shrubland communities since the middle of 20th century [27], [28]. These practices include removal and control of woody species, seeding with forage species, and grazing management [28]–[30]. Various techniques, including controlled burning, herbicide, and mechanical treatments, have been implemented to force type conversion from a community of woody vegetation to a stable and persistent community of herbaceous vegetation, or to at least postpone succession [27], [31]. It has been shown that suitable soil, climate, and topography are necessary for successful type conversion for range use [27], [28]. While periodic repeat burning has been used to reduce shrubby vegetation in diverse locations, in California it is considered the least effective method for removing woody vegetation for range improvement [27]. Despite extreme treatments and follow-up activities, chaparral can be very persistent, and the intended long term type conversion with the objective of range improvement can be difficult to achieve [28], [31]. Thus, contrary to the hypothesis that repeat short interval fires will cause type conversion, past range improvement studies present an alternative hypothesis that type conversion may be difficult and should be a relatively rare phenomenon across the landscape.

To date, most studies of potential type conversion of chaparral by repeat fire have been implemented at the local-site scale over short time intervals (e.g. Keeley and Brennan [13]). Patterns demonstrated at these small scales may not be persistent over time or across larger regional scales. Due to the large-scale spatial and temporal variability of wildfire disturbance and post-fire response in terrestrial ecosystems, various studies have utilized remote sensing data to monitor and study post-fire vegetation response and recovery trajectories [17], [32]–[36]. Remote sensing techniques offer the opportunity to study fire effects and recovery dynamics across large areas, providing measurements at the landscape (local-to-regional) scale. To evaluate the effects of short fire return intervals on chaparral recovery across coastal southern California, we used Landsat-5 Thematic Mapper (TM) images and a remotely sensed vegetation index. The effects from two fires in rapid succession (fire return intervals of less than 8 years) were determined by comparing twelve areas that burned twice to adjacent control areas burned only once. We used our results to examine two alternative hypotheses: 1. Two fires separated by a short interval should adversely impact the recovery of obligate seeding species following the second fire, thus reducing chaparral cover in comparison to an adjacent area burned only once; 2. Persistent type conversion of chaparral shrubland to grassland is rare and spatially limited, even after short return interval fires (<8 years). Correlations between vegetation recovery and climate, fire history and elevation variables were also examined.

## Methodology

### Study region

**Figure 1:**
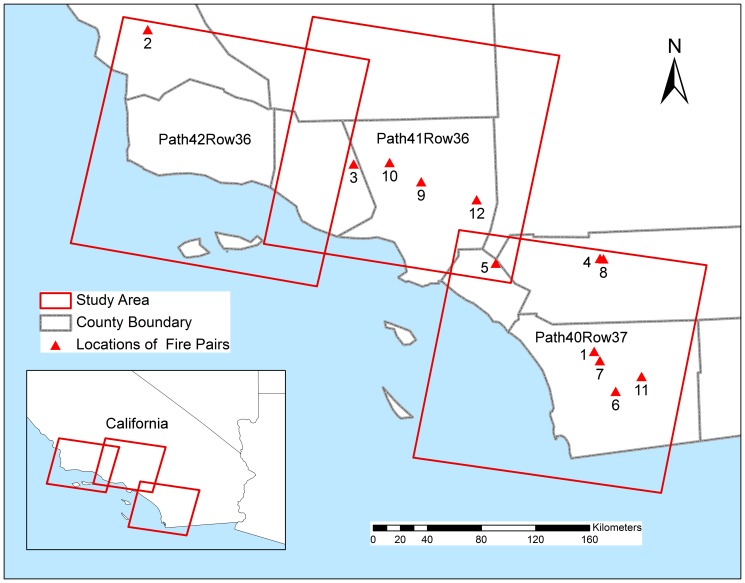
Study area. Study area covering coastal southern California. Path and row are indicated in the center of each Landsat TM scene. Locations and ID numbers of 12 example fire pairs are indicated on the map.

The study area is located in coastal southern California within the combined area of three Landsat-5 TM scenes (path 40, row 37; path 41, row 36; path 42, row 36; Figure 1). Patchy mosaics of grassland, woodland, coastal sage scrub, and chaparral are the dominant vegetation types in the study area [37]. The Mediterranean climate is characterized by cool-wet winters (December to February) and dry-hot summers (June to September). Large fires occur predominantly from mid-summer until November, corresponding to low live fuel moisture [38] and the occurrence of Santa Ana winds [39]. While historic fire return intervals are poorly known, it is generally believed that they varied from 20–65 years depending on aspect, elevation and distance to coast [40], [41].

### Data

Landsat-5 TM imagery with a 30 m spatial resolution covering the study area in 1985 and 2010 was obtained from the USGS GLOVIS website (http://glovis.usgs.gov; Table 1). September dates were targeted for all imagery to ensure phenological comparability. ACORN (Atmosphere Correction Now; http://www.imspec.com) was selected to implement reflectance retrieval for the 1985 scene in each path-row, due to lower cloud cover for these scenes. The 2010 scenes were then calibrated to the 1985 scenes using pseudo-invariant features found by iMAD (iteratively re-weighted Multivariate Alteration Detection) [42].

**Table 1:** Dates and estimated cloud cover for each Landsat image used in the study.

Path	Row	Date	Estimated Cloud Cover (%)
40	37	14 Sep 1985	0
40	37	19 Sep 2010	23
41	36	21 Sep 1985	0
41	36	26 Sep 2010	0
42	36	12 Sep 1985	0
42	36	01 Sep 2010	33

Because pre-fire chaparral areas were targeted in this analysis, a map of vegetation type coincident with or prior to 1985 was needed. The 1977 CALVEG (Classification and Assessment with Landsat of Visible Ecological Groupings) —a statewide classification system developed by US Forest Service— was used to subset the Landsat imagery. The 1977 CALVEG map was created based on 1∶250,000 scale Landsat Multispectral Scanner (MSS) imagery acquired between 1977 and 1979. Through identifying distinctions among canopy reflectance values of Landsat MSS imagery, field verification and professional guidance, vegetation type “series” based on dominant overstory species were mapped. The Minimum Mapping Unit (MMU) of the 1977 CALVEG is 162 ha (400 acres), mainly due to the limitations of image resolution (http://portal.gis.ca.gov/). MMU refers to the smallest size or dimensions for an entity to be mapped as a discrete feature for a given map scale [43].

Historical fire perimeters between 1985 and 2010 were derived from California Department of Forestry and Fire Protection FRAP (Fire and Resource Assessment Program; http://frap.cdf.ca.gov/) and US Forest Service/US Geological Survey MTBS (Monitoring Trends in Burn Severity; http://www.mtbs.gov/) datasets. Updated annually, the CALFIRE FRAP dataset represents the most complete GIS-format record of fire perimeters in California dating back to the early 1800s. This dataset provides extensive information for the period 1985–2010. The CALFIRE FRAP MMU is not consistent with the MMU for the CALVEG dataset, and varies by location and through time. During the period 1950–2001, the CALFIRE FRAP dataset included US Forest Service fires larger than 4 ha (10 acres) and CALFIRE fires larger than 121 ha (300 acres). Bureau of Land Management and National Park Service data were added starting in 2002, and the MMU for CALFIRE fires dropped to 20 ha (50 acres) for shrubland and 4 ha (10 acres) for forest. The variable MMU for this dataset is an acknowledged weakness, but the CALFIRE FRAP dataset should still capture most of the area burned by wildfires in southern California during the study period. MTBS data were used to supplement the CALFIRE FRAP dataset. MTBS aims to map perimeters and burn severity of fires larger than 405 ha (1,000 acres) across the western United States using 30 m Landsat TM data [44].

Climate data—monthly mean precipitation and minimum temperature—between 1985 and 2010 were downloaded from the PRISM (Parameter-elevation Regressions on Independent Slopes Model) website (http://www.prism.oregonstate.edu/). PRISM makes use of point measurements of precipitation and temperature to generate continuous digital grid estimates of climatic data with a 4 km spatial resolution [45]. The DEM (Digital Elevation Model) used in this study was derived from the SRTM (Shuttle Radar Topography Mission; http://www2.jpl.nasa.gov/srtm/) with a 30 m spatial resolution consistent with the Landsat-5 TM imagery. Climatic and topographic variables derived from PRISM and SRTM were used in linear regression as explanatory factors of vegetation recovery following short-interval fires.

### Analysis procedure

**Figure 2:**
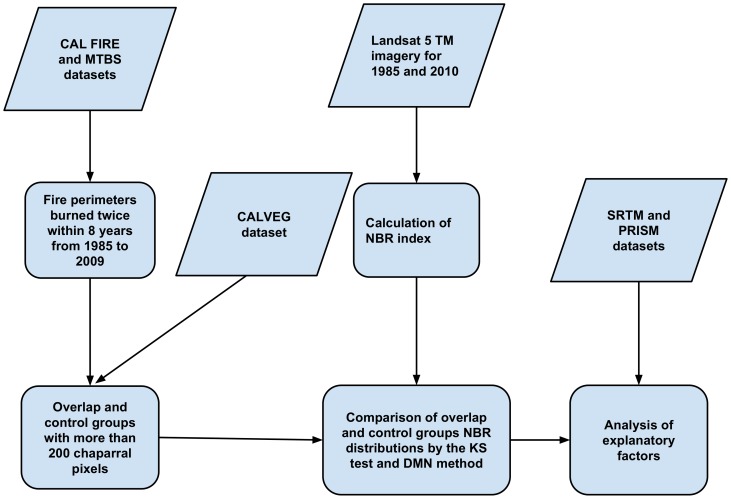
Flowchart of the analysis procedure.

Figure 2 illustrates the analysis procedure we used. Landsat-5 TM data were used for calculating NBR (Normalized Burn Ratio-described below) values for both 2010 and 1985 images. Conditional historical fire perimeters were extracted from the fire history dataset, in order to determine areas that burned twice (overlap) from October 1985 to December 2009 with a short fire return interval (defined here as <8 years) during this period, and similar areas that had only burned once in the latter of the two fires (control). Differences in the NBR distributions between overlap and control groups were used to compare vegetation recovery; conditions affecting the localized vegetation vigor or phenology in the control and overlap areas are assumed to be similar. Lastly, variables that could potentially influence vegetation recovery, including elevation, post-fire temperature, and post-fire precipitation, were compared to changes in NBR distributions using scatter plots and linear regression. A total 1804 fire cases were recorded in the CALFIRE FRAP datasets between 1984 and 2009 within the study area. Before analyzing the effects of explanatory factors on potential type conversion, global Moran's I was calculated to determine whether the change in DMN values (Table 2-described below) were spatially independent [46]. Moran's I measures the spatial autocorrelation based on feature locations and associated variable values.

**Table 2:** Descriptions of indices used in this study.

Indices	Calculations	Indications
D_1985_	The maximum difference between overlap and control groups in NBR cumulative distributions from the 1985 image	Difference in vegetation cover between a control area and a corresponding overlap area in 1985
DMN_year_	Median (Control(NBR_year_)) – Median (Overlap(NBR_year_))	Higher (positive)/lower (negative) vegetation cover within a control area relative to a corresponding overlap area in a specific year
Change in DMN	DMN _2010_ – DMN _1985_	Increase (positive)/decrease (negative) in vegetation cover within a control area relative to a corresponding overlap area after repeat burn

#### Calculation of NBR

Various remote sensing instruments and techniques have been used for evaluating wildfire and its impacts [2]. It has been shown that bands 3, 4, 5, and 7 from Landsat-5 TM data have the largest responses to burn severity [47], [48]. NBR uses a combination of band 4 (near infrared or NIR) and band 7 (shortwave infrared or SWIR) that provides the best distinction between burned and unburned areas. NBR is calculated as: 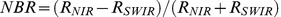
(Equation 1)


where R_NIR_ and R_SWIR_ represent surface reflectance for TM band 4 (760–900 nm) and band 7 (2,080–2,350 nm). NBR is functionally identical to NDII (Normalized Difference Infrared Index), a SWIR-based vegetation index correlated with vegetation water content [49]. Changes in NBR and its relative form (relative differenced Normalized Burn Ratio (RdNBR)) derived from Landsat data have been widely demonstrated to correlate with field measurements of burn severity [48], [50]. In this study, NBR is used as an indicator of vegetation cover, where an increase in NBR value is correlated with higher vegetation cover [48]. Lopez Garcia and Caselles [51] found that NBR not only can be used for the discrimination of burned areas, but was also capable of monitoring post-fire regeneration over burned areas in a Mediterranean ecosystem. In a more recent study, NBR was also found to support longer detectable periods of fire effects than other common band ratios, such as NDVI (Normalized Difference Vegetation Index) and EVI (Enhanced Vegetation Index) [52]. NBR was calculated using Equation 1 [48] for all 1985 and 2010 Landsat-5 TM images.

#### Comparison of fire overlap and control groups

Fires that burned the same area twice within a period of eight years were selected for analysis. This time frame was chosen based on time required for obligate seeding shrub species to begin producing seed [8], [53], [54]. All successive fires with short return intervals (<8 years) within the study area, from October 1985 to December 2009, were selected. Some apparently incorrect fire perimeters in the CALFIRE dataset were deleted from the final result. In order to remove any edge effects, all fire perimeters were buffered by 3 pixels (90 m). The perimeters of successive fires from CALFIRE FRAP were replaced by MTBS where fire size exceeded the size necessary for inclusion in MTBS. MTBS burn severity was then used to exclude unburned islands inside of fire perimeters. Finally, any areas burned in the 8 years preceding the 1985 Landsat image, as indicated in the CALFIRE FRAP dataset, were excluded to ensure unlikely change in vegetation type since the 1977 CALVEG classification.

For each fire pair, NBR values of pixels classified as chaparral in the 1977 CALVEG classification and within the fire overlap area were derived from 1985 and 2010 imagery to constitute groups called “overlap”. Similarly, NBR values within the latest (second) fire area, but excluding the overlap area, were also derived to constitute groups called “control”. The overlap group contains NBR values of chaparral pixels burned twice, and the control group contains NBR values of chaparral pixels burned once since 1985 by the most recent fire of the two fires that burned the “overlap” group. The MMU inconsistency of CALFIRE FRAP dataset in the study period, as well as the difference in MMU between 1977 CALVEG classification and CALFIRE FRAP dataset, added uncertainty to the comparison of fire overlap and control groups.

**Figure 3:**
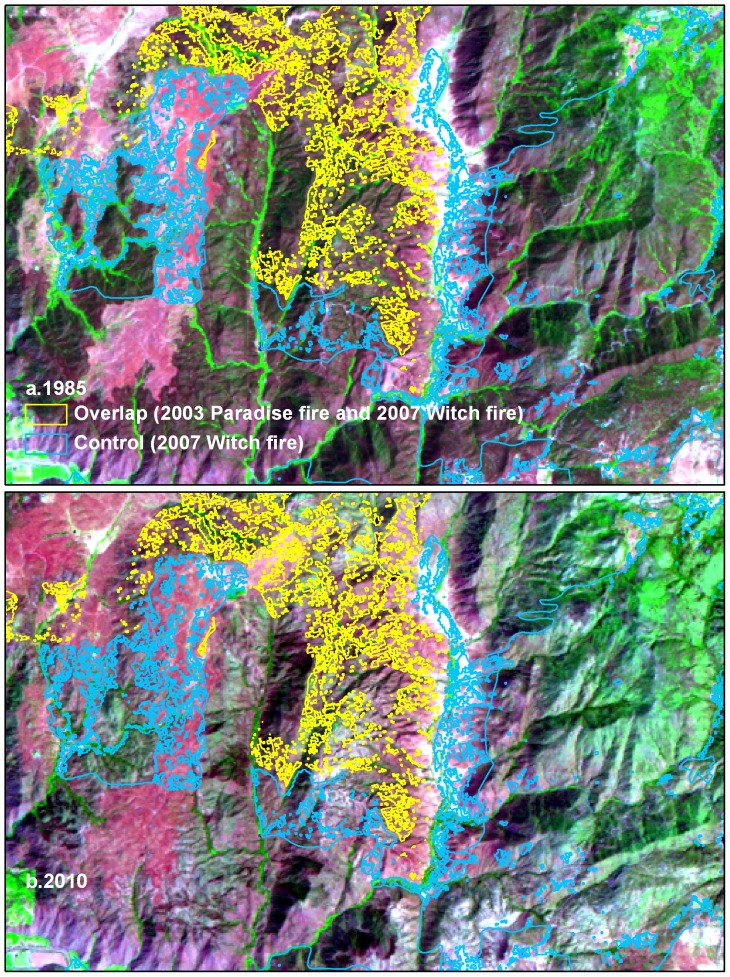
Fire areas and 1985 and 2010 Landsat images. Landsat-5 TM images covering areas burned by the Cedar fire in 2003 and the Witch fire in 2007. a. 1985 Landsat-5 TM, bands 5, 4, and 3 displayed as red, green, and blue, respectively. b. 2010 Landsat-5 TM, bands 5, 4, and 3 displayed as red, green, and blue, respectively.

Figure 3 shows an example of qualified fire perimeters within the Landsat-5 TM imagery before and after the repeat burn. Targeted chaparral pixels within fire perimeters were not required to be spatial contiguous, due to the multiple masks applied to the fire perimeters. A minimum pixel cluster size was not used to exclude isolated pixels from the analysis, since further analysis was based on the distribution of all pixels belonging to the control or overlap groups.

Distributions of NBR values in the overlap and control pairs with more than 200 chaparral pixels each were compared in two ways: the KS (Kolmogorov-Smirnov) test and a DMN (Difference Median NBR) method. The threshold of 200 pixels was established based on obtaining a population sufficient for calculating a distribution of values for comparison, producing 39 potential overlap and control pairs. In order to make sure that the difference between overlap and control distributions in 2010 was likely due to repeat fires, and not differences in pre-fire vegetation cover, only the fire pairs with similar 1985 pre-fire NBR distributions were further compared. The KS test is a non-parametric test for comparing differences between two distributions. The D value returned by the KS test measures the maximum difference in two cumulative distributions [55]. In this study, the D value is the maximum difference between two cumulative distributions of NBR values in the pixels derived from control and overlap pairs (Table 2). Small D values calculated for 1985 indicated that pre-fire vegetation cover distributions were similar for the overlap and control areas. As D_1985_ values increased, pre-fire vegetation cover distributions were more distinct.

A threshold D_1985_ value of 0.16 was empirically established to identify pre-fire NBR distributions that were similar enough for post-fire comparison. This threshold was determined based on visual inspection of NBR distributions, and on correlations between D_1985_ and the difference in median NBR values, a measure described below. A local maximum in this correlation was found for a D_1985_ value of 0.16. The D_1985_ constraint produced 12 pairs of control and overlap areas with similar pre-fire distributions of NBR values and at least 200 chaparral pixels in both groups.

DMN was calculated using the equation in Table 2, taking the difference between median NBR values of control and overlap distributions. DMN is a measure of difference between control and overlap distributions, but unlike D value, DMN by definition is based on the center of the distribution and can be positive or negative. A positive DMN value indicates a higher median vegetation cover in a control area relative to an overlap area; a negative value indicates a higher median vegetation cover in an overlap area relative to a control area. DMN values should be strongly positive in 2010 relative to 1985 if type conversion occurred at the landscape scale, due to reduced vegetation cover in areas burned twice.

#### Analysis of explanatory factors

The potential explanatory factors listed in Table 3 were compared to changes in DMN values from pre-fire (1985) to post-fire (2010) using simple linear regression. For fire history factors, scatter plots were made to show the relationships between time interval between the two fires, time since second fire to 2010 (e.g. time for recovery), and change in DMN values. For each fire pair, the mean elevation, mean precipitation in the wet season (winter and spring) immediately following fire, and mean average January minimum temperature one year following fire were calculated from the PRISM and SRTM datasets (Table 3). Mean elevation, mean precipitation in the wet season (winter and spring), and mean average January minimum temperature were compared to change in DMN to analyze potential trends.

**Table 3:** Explanatory variables used in simple linear regression analysis.

Explanatory Variables	Descriptions
Time since second fire to 2010	Time since the occurrence of second fire in the short interval fire pair to 2010 (years)
Time interval between two fires	Interval between successive fires in fire pair (years)
Mean precipitation in winter season	Mean precipitation within area burned by second fire in December, January and February immediately following fire (mm)
Mean precipitation in spring season	Mean precipitation within area burned by second fire in March, April and May immediately following fire (mm)
Mean elevation	Mean elevation within area burned by second fire (m)
Mean average January minimum temperature	Mean average minimum temperature within area burned by second fire, in January following second fire (C)

#### Validation of the DMN comparison

One hundred points were generated randomly in the both overlap and control areas of each qualified fire pair. High resolution imagery in Google Earth was used to inspect the vegetation cover at each point. The used Google Earth image dates ranged from 2009–2013, in order to use an image date that covered both the control and overlap areas in each pair and provide the highest contrast between evergreen chaparral canopies and senesced herbaceous vegetation or drought deciduous shrubs. Each randomly generated point was labeled as having evergreen shrub canopy or non-evergreen shrub canopy (e.g. grass, drought-deciduous shrub, soil). The percentages of evergreen shrub canopy pixels within the control and overlap groups were calculated, and differences between the control and overlap groups were compared to DMN_2010_.

## Results

### Comparisons

Twelve of 39 fire pairs were sufficiently similar in the pre-fire (1985) distribution of NBR values (Table 4) to be considered adequate comparisons. The global Moran's I result (Moran's I index: 0.104; z-score: 1.07; p-value: 0.28) on the change in DMN indicates that values are spatially independent, likely due to the reduced degree of pixel contiguity caused by complex overlapping boundaries of chaparral polygons and burned areas (Figure 3).

**Table 4:** Comparison of results for fire pairs with similar cumulative distributions of 1985 NBR values^a^.

ID Number	Path Row	Year1 Fire1	Year2 Fire2	D_1985_	Change in DMN (2010–1985)	Overlap Area (ha)
1	p40r37	2003 Paradise	2007 Poomacha	0.135	0.045	1031
2	p42r36	1996 Highway58	2003 Parkhill	0.078	0.037	40
3	p40r37	2003 Piru	2007 Ranch	0.065	0.025	79
4	p40r37	2001 Silent	2006 Esperanza	0.140	0.017	22
5	p40r37	2002 Green	2006 Sierra	0.128	0.016	214
6	p40r37	2003 Cedar	2007 Witch	0.056	0.009	2130
7	p40r37	2003 Paradise	2007 Witch	0.057	−0.001	1518
8	p40r37	1999 Pine	2006 Esperanza	0.066	−0.005	210
9	p41r36	2007 North	2009 Station	0.086	−0.018	60
10	p41r36	2002 Copper	2007 Buckweed	0.060	−0.019	1368
11	p40r37	1999 Banner	2002 Pines	0.110	−0.051	23
12	p41r36	1996 Bichota	2002 Curve	0.094	−0.095	25

ID number refers to fire pairs shown in Figure 1.

a Sorted by decreasing change in DMN.

**Figure 4:**
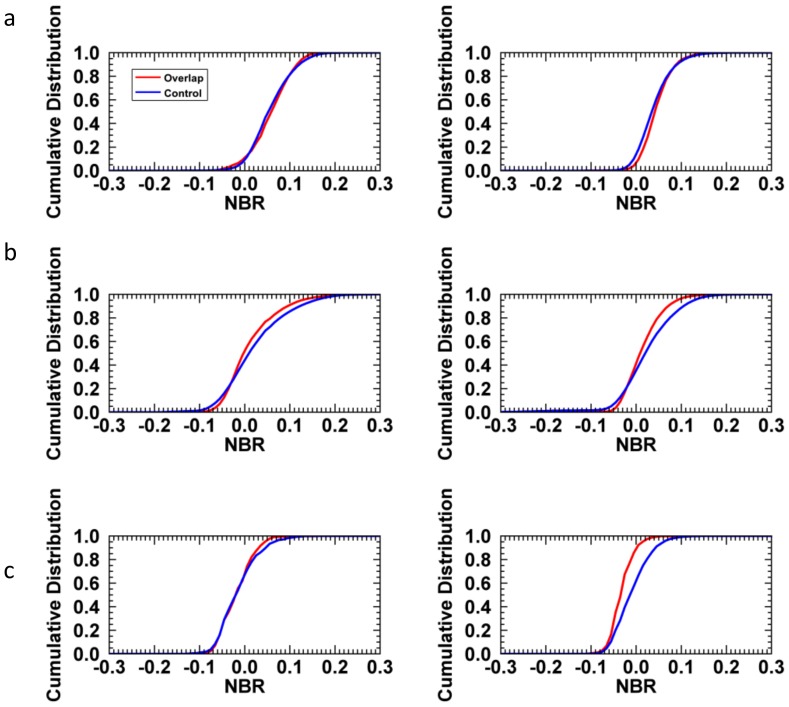
Pre-fire (1985) and post-fire (2010) cumulative distributions. Pre-fire (1985) and post-fire (2010) cumulative distributions of NBR values from different fire pairs. a. 1999 Pine fire and 2006 Esperanza fire b. 2003 Cedar fire and 2007 Witch fire. c. 1996 Highway58 fire and 2003 Parkhill fire.

All 12 control-overlap pairs were further compared using their NBR cumulative distributions. Control-overlap pairs generally had similar distributions in 1985 and 2010, but there were differences in the distributions between specific pairs. For example, for the 1999 Pine fire and 2006 Esperanza fire, the NBR distribution for the overlap area shifts to slightly higher median NBR than the control area in 2010 (Figure 4a). By contrast, for the 2003 Cedar fire and 2007 Witch fire, the difference between control and overlap distributions becomes smaller after being burned twice (Figure 4b). For the 1996 Highway58 fire and 2003 Parkhill fire, the overlap and control group distributions were more similar in 1985, but in 2010 there is a larger difference between them, with the overlap group shifted to lower NBR (lower vegetation cover) than the control group (Figure 4c). Table 4 shows that most of fire pairs did not undergo substantial changes in DMN. Six of twelve cases, representing a total area of 3515 ha (52% of the burned area evaluated), have a positive change in DMN consistent with our hypothesis that repeat short interval fire should cause type conversion. Six of twelve cases have a negative change in DMN, and the most extreme changes in DMN have negative values.

### Validation results

**Figure 5:**
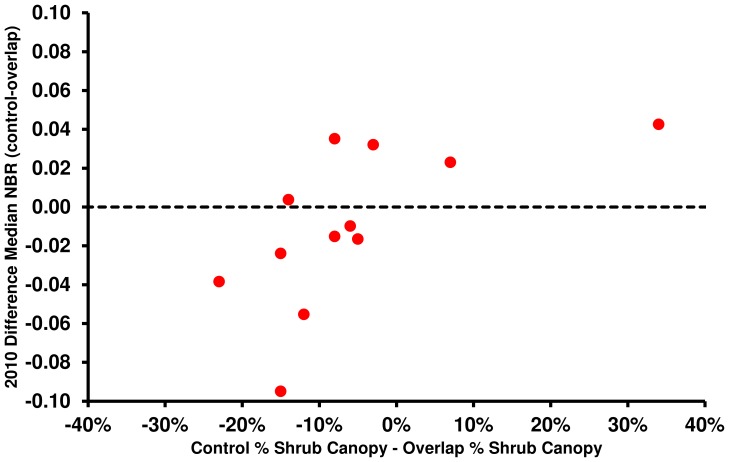
Post-fire difference in percent evergreen shrub canopy versus DMN_2010_. Post-fire difference in percent evergreen shrub canopy, derived from Google Earth imagery for control and overlap areas, versus DMN_2010_.

Difference in percent evergreen shrub canopy cover between control and overlap areas, calculated from Google Earth imagery, was compared to DMN_2010_ (Figure 5). The two measures have a significant, positive correlation (Pearson's correlation: 0.63, p-value: 0.03), indicating that larger differences in evergreen shrub cover between control and overlap areas are correlated with larger differences in DMN calculated from 30 m Landsat-5 TM data.

### Fire history factors

**Figure 6:**
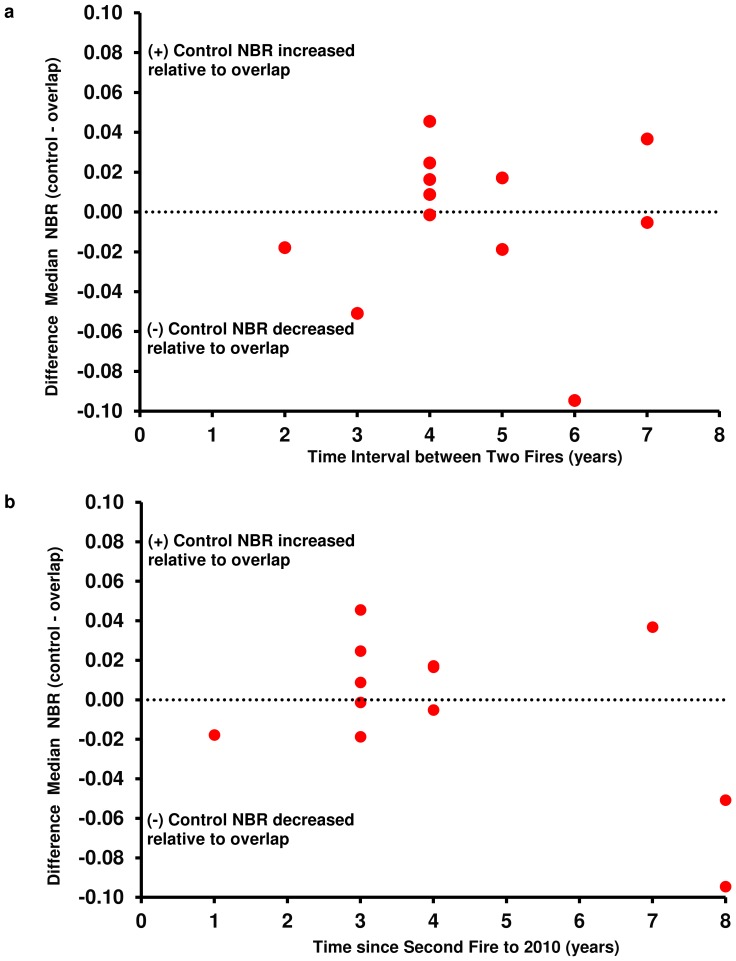
Time interval and time since second fire versus change in DMN. **a.** Time interval between two fires versus change in DMN from 1985 to 2010. **b.** Time since second fire to 2010 versus change in DMN from 1985 to 2010.

Time interval between two fires and time since second fire to 2010 were plotted against change in DMN (Figure 6a; Figure 6b). There was no apparent trend between time interval between fires or recovery time and change in DMN (Table 5). Shorter fire return intervals did not produce higher DMN values, which would be consistent with type conversion due to elimination of obligate seeding species.

**Table 5:** Simple linear regression results for explanatory variables.

Variables	Pearson's r	R-squared	P_r_^a^(>|t|^ b^)
Time since second fire to 2010 (years)	−0.49	0.24	0.106
Time interval between two fires (years)	0.02	0.0004	0.963
Mean precipitation in winter season (mm)	−0.02	0.0004	0.963
Mean precipitation in spring season (mm)	−0.02	0.0004	0.963
Mean elevation (m)	−0.80	0.64	0.0019*
Mean average January minimum temperature (C)	0.02	0.0004	0.963

* P_r_ <0.01; ^a^ Probability of a standard normal variable; ^b^ Value of t distribution.

### Climatic and topographic factors

**Figure 7:**
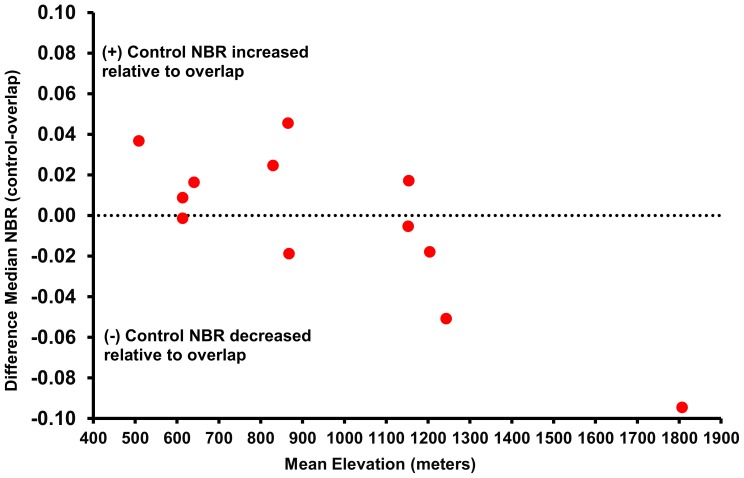
Mean elevation versus change in DMN.

Correlation analysis of change in DMN with mean post-fire precipitation in wet season and mean average minimum temperature in January for each fire pair revealed no trends and is thus not shown here (Table 5). Elevation, by contrast, was most strongly correlated with the change in DMN between 1985 to 2010: as elevation increased, the change in DMN values from 1985 to 2010 tended to be more negative (Figure 7; Table 5).

## Discussion

This remote sensing-based approach allows evaluation of the effects of short fire return interval on chaparral recovery on broader spatial and temporal scales than previous site-scale studies. This study evaluated change at a relatively coarse spatial resolution (30 m), and thus our method could not detect changes in species composition within chaparral. Positive changes in DMN, indicative of possible reduction in vegetation cover, were found for 50% (6 out of 12) of the sites examined. Only two of these six overlap/control areas (16% of the burned area evaluated) at lower elevation showed large changes in DMN that are consistent with type conversion. In short, our evidence does not support that extensive type conversion has occurred on the landscape scale due to recent short-interval fires. Hence our results are consistent with the hypothesis that it is difficult to convert existing chaparral to grassland [28], [31].

Replacement of specific shrub species with other shrub species may result in the same vegetation type and similar vegetation cover. This type of species-level change cannot be ruled out based on our landscape scale analysis. Also, use of median NBR as a metric does not exclude changes in the tails of NBR distributions that may be attributable to type conversion. However, distribution plots (Figure 4) did not indicate any systematic changes in the qualitative shape of NBR distributions. In addition, our methodology excluded cumulative, small scale type conversions that are beyond the capacity of Landsat-based analysis, and did not examine areas burned more than twice during the study period. It is also possible that locations most sensitive to long-term type conversion have already been converted many decades ago, so it may be difficult to discern which shrub species or topographic locations have suffered the most substantial losses.

Elevation has been deemed as one of the strongest predictors of post-fire regrowth patterns in chaparral [14], [16]. In this study, simple linear regression also suggested that elevation explained 64% of the variability in change in DMN and produced the strongest correlation (−0.80). As precipitation increases with elevation, non-sprouting species become less common [12]. As multiple burns can reduce non-sprouting species more significantly [13], [21], overlap areas at higher elevation could be expected to be less susceptible to type conversion than overlap areas at lower elevation. The invasion of alien plants in chaparral has been also attributed to the proximity of chaparral to coastal sage scrub and herbaceous communities at lower elevations with gentle slopes [17], [21], [56]. It is also likely that low elevation sites were more vulnerable to human activities, such as urbanization and increased ignition frequency.

Contrary to previous studies, effects of time interval between two fires and time since second fire were not found to be significant for explaining variability in post-fire recovery [17]. The apparently strong influence of elevation on differences in recovery between once- and twice-burned areas may have obscured more subtle differences in recovery caused by time interval or time since second fire. Also, reliance on distributions calculated from large areas prevented analysis of other factors that may influence recovery, including topographic slope and aspect, and soil type. Post-fire recovery of chaparral is likely to be sensitive to soil moisture, and moisture availability can be affected by topographic exposure [17]. Fire intensity and duration also were not incorporated to explain the variability in change in DMN. Nevertheless, impacts of fire intensity and duration on vegetation recovery are complicated in chaparral; for some species, fire inhibits seeding recruitment or delays resprouting recruitment, but in other species fire enhances plant recovery [14].

Fire-adaptive traits enable chaparral species to reestablish themselves rapidly even following severe stand-replacing fire, referred to as auto-succession or a direct regeneration process first defined by Hanes [12]. Auto-succession assumes chaparral species are highly adapted to disturbance, and the phenomenon of vegetation type conversion (losing plant species) after fire is rare [57]; nevertheless, an extreme change in plant composition may occur when fire disturbance exceeds its natural range of variation [58]. In our study, most burned areas with short fire return interval demonstrated similar post-fire response to control areas with longer fire return intervals (> 8 year). Compounding effects of multiple disturbances may still trigger type conversion in chaparral, but this process may be rare and spatial-limited.

While multispectral remote sensing is insensitive to compositional shifts within shrubland vegetation, it may be able to complement future site-scale studies of fire recovery and be used to understand fire recovery processes occurring across large regions. Past studies using hyperspectral and very high spatial resolution remote sensing have demonstrated that dominant shrubland or forest species as well as main post-fire regeneration classes can be mapped [59]–[61]. Advanced image analysis techniques (i.e. object-oriented image analysis) may also potentially improve the accuracy of post-fire related information [62]. Current and proposed satellite missions providing improved fire detection and monitoring [63], [64] and mapping of vegetation functional types [65] may allow further enhancements to monitoring of vegetation response to wildfire at continental-to-global scales.

## Conclusions and Future Work

Using a historical wildfire dataset and remote sensing imagery, we have analyzed differences in vegetation cover following short interval fires across a broad expanse of southern California chaparral shrublands. Changes in the distributions of NBR values from qualified overlap/control fire pairs did not indicate extensive type conversion of shrubland to grassland, suggesting that type conversion of shrubland to grassland may be a spatially limited and regionally rare phenomenon. Sites with large, positive changes in DMN values most consistent with type conversion were at lower elevation in proximity to communities of coastal sage scrub, in accordance with previous results found by Lippitt *et al.* [17]. Simple linear regression indicated that only elevation was strongly correlated to observed variation in vegetation recovery across the once and twice burned pairs. At lower elevation, non-sprouting species are more common and thus the vegetation is likely more vulnerable to type conversion [12], [14], [17].

While loss of native shrublands is an important conservation issue, our findings suggest that recent localized studies demonstrating shrub declines should be resurveyed through time. Due to variability in chaparral recovery from short return interval fires, conservation strategies and management plans may need to take into account elevation and other site characteristics. Under future climate change scenarios in the western United States, a small change in climate could possibly lead to drastic and divergent shifts in fire activity and ability of chaparral species to recover from fire, with the potential of reaching “tipping points” of type conversion in Mediterranean-type ecosystems [66]. Prospective management plans should be prepared to deal with this situation. In addition, more studies are needed to investigate disturbance-chaparral interactions by quantifying the relative contributions of varying fire frequency on the composition of chaparral species.
